# CD5 on dendritic cells regulates CD4+ and CD8+ T cell activation and induction of immune responses

**DOI:** 10.1371/journal.pone.0222301

**Published:** 2019-09-06

**Authors:** Hui Li, Erica Burgueño-Bucio, Shin Xu, Shaonli Das, Roxana Olguin-Alor, Craig A. Elmets, Mohammad Athar, Chander Raman, Gloria Soldevila, Hui Xu

**Affiliations:** 1 Department of Dermatology, University of Alabama at Birmingham. Birmingham, Alabama, United States of America; 2 Department of Immunology, Instituto de Investigaciones Biomédicas, Universidad Nacional Autónoma de México, Ciudad de México, Mexico; 3 Department of Medicine, University of Alabama at Birmingham. Birmingham, Alabama, United States of America; Uniformed Services University of the Health Sciences F Edward Hebert School of Medicine, UNITED STATES

## Abstract

The role of CD5 as a regulator of T cell signaling and tolerance is well recognized. Recent data show expression of CD5 on different subtypes of human dendritic cells, however its functional relevance in modulating DC mediated responses remains poorly understood. In this study, we show CD5 is expressed on CD11c+ DC from murine thymus, lymph node, spleen, skin and lung. Although the development of DC subpopulations in CD5^-/-^ mice was normal, CD5-deficient DC produced significantly higher levels of IL-12 than wild type DC in response to LPS. CD5^-/-^ DC, in comparison to CD5^+/+^ DC, enhanced the activation of CD4+ and CD8+ T cells *in vitro* and *in vivo and* induced significantly higher production of IL-2 and IFN-gamma by T cells. Consequently, CD5^-/-^ DC were significantly more potent than wild type DC in the induction of anti-tumor immunity and contact hypersensitivity responses in mice. Restoration of CD5 expression in CD5^-/-^ DC reduced IL-12 production and inhibited their capacity to stimulate T cells. Collectively, these data demonstrate that the specific expression of CD5 on DC inhibits the production of inflammatory cytokines and has a regulatory effect on their activity to stimulate T cells and induce immune responses. This study reveals a previously unrecognized regulatory role for CD5 on DC and provides novel insights into mechanisms for DC biology in immune responses.

## Introduction

CD5 is a 67 kDa type 1 cell surface protein with a large cytoplasmic domain containing multiple potential phosphorylation sites that recruit regulators of T cell signaling [1, 2]. CD5 is expressed by thymocytes, mature T cells and the B1a subset of B cells [3]. CD5 regulates TCR signaling, tunes threshold for T cell activation during thymocyte development [4–7] and suppresses the activation of peripheral T cells through inhibition of TCR-proximal signaling in the immunological synapse [8, 9]. Increased CD5 expression on T cells is associated with a lower response to antigen stimulation and immunity [10–12]. A high expression of CD5 on T-cells is involved in the induction of tolerance and generation of Treg cells [13–16]. In contrast, lack of CD5 mediated signals in T cells leads to hyper-activation and increased activation induced cell death [17, 18]. Changes in CD5 expression also effects development and function of CD5+ B1a cells [19]. The function of CD5 in lymphocytes has been extensively studied, however, its role in other immune cell populations is largely unknown.

Dendritic cells (DC) depending on their state of differentiation and/or maturity play a central role in both induction and regulation of immune responses [20–25]. In an immune response, DC produce IL-12, a cytokine essential for Th1 differentiation and IL-23 that promotes stability and pathogenicity of Th17 cells [26, 27]. In a normal immune response, Th1 and Th17 have important roles in the protection against infectious diseases and cancers; however, dysregulation and loss of tolerance promotes their conversion to pathogenic autoreactive T cells [28–30]. Thus, the regulation of cytokine production by DC is necessary for homeostasis in immunity. Recent reports show that a subpopulation of human or rat dendritic cells (DC) express CD5 mRNA [31, 32]. In human, conventional DC type 2 (cDC2) from tonsils, lymph node (LN) and blood can be further classified on differential expression of CD5 [33]. T cell proliferation and cytokine production varies with expression levels of CD5 on human blood plasmacytoid DC (pDC) and human skin Langerhans and dermal DC [34, 35]. However, it remains unclear whether CD5 is only a marker for different DC subsets or it has a role in DC function.

In the current study, we characterized the expression of CD5 on murine DC in lymphoid and non-lymphoid organs and investigated whether CD5 regulates the function of DC in the activation and differentiation of T cells and in the induction of immune responses. We found that CD5 is commonly expressed on murine DC and has an inhibitory effect on the ability of DC to stimulate CD4 and CD8 T cells and to induce anti-tumor and contact hypersensitivity responses. CD5-dependent regulation of IL-12 production by DC is a mechanism for DC mediated regulation of T cells and immune responses.

## Materials and methods

### Mice

CD5^-/-^, TCR transgenic OT-II and OT-I mice, wild-type (WT) C57BL/6 and ^-/-^and WT Balb/c mice (Jackson Laboratory (Bar Harbor, ME) and 2D2 mice [36] were used in this study. TCR transgenic OT-II and OT-I are specific for ovalbumin with restriction for MHCII and MHCI, respectively. 2D2 TCR transgenic TCR have MHCII restricted specificity for myelin oligodendrocyte glycoprotein peptide 35–55 [36, 37]. Male and female mice were used. C57BL/6 and Balb/c mice were utilized in the study to enhance the rigor and to show that CD5 effects were not dependent on mouse strains. All animal procedures were performed according to National Institutes of Health guidelines and were approved by the Institutional Animal Care and Use Committee of the University of Alabama at Birmingham and by the Animal Experimental Bio-Ethics Guidelines from the ‘‘Comité para el Cuidado y Uso de Animales de Laboratorio (CICUAL)” of the Instituto de Investigaciones Biomédicas, Universidad Nacional Autónoma de México (Protocol #108).

### Purification of DC from tissues and generation of bone marrow derived dendritic cells

Spleen (SP), lymph node (LN), thymus and lungs were digested with collagenase D (0.5mg/ml) and DNAse (25U/ml) in 5% FCS RPMI media at 37°C for one hour. Tissues were mechanically disaggregated. Shaved skin was removed and incubated in medium with dispase (25 mg/ml) at 37°C for 1–1.5 hours. Epidermis was peeled from the dermis and incubated in medium with 2.5% Trypsin, 1mM Ethylenediaminetetra-acetic acid (EDTA) and DNAse (25 U/ml) at 37°C for 1.5 to 2 hours. The dermis was incubated in medium with collagenase D (0.5 mg/ml) and DNAse (25 U/ml) at 37°C for 1 hour. Cell suspensions were used for staining and analysis of DC. CD11c+ DC from LN and SP were purified by using anti-CD11c antibody coupled MACS beads according to the manufacturer’s instruction (Miltenyi Biotec., Auburn, CA). For *in vitro* stimulation of DC, CD11c+ DC were cultured for 24 hours in the presence or absence of 0.1μg/ml Lipopolysaccharide [(LPS) Sigma, St. Louis, MO].

Bone marrow derived dendritic cells (BMDC) were generated from bone marrows of WT C57BL/6 and CD5^-/-^ mice as previously described [38]. Briefly, bone marrow cells were extracted from the femurs and tibias and cultured in 10% Fetal Calf Serum (FCS) RPMI1640 media supplemented with 10 ng/ml of recombinant Granulocyte-macrophage colony-stimulating factor [(GM-CSF) BD Bioscience] and 10 ng/ml IL-4 (Sigma) at 1x10^6^cells/ml. LPS (1 μg/ml) was added to stimulate maturation of BMDC at day 5 and BMDC were harvested 24 hours later. Around 95% cells expressed DC marker CD11c.

### Flow cytometry analysis

The analysis of DC and T cells by flow cytometry was conducted as described in our previous studies [39] and resident and migratory DC in SP and LN have been gated and analyzed as previously reported [40, 41]. Briefly, DC from SP and LN or BMDC were incubated with an anti-CD16/CD32 antibody (2.4G2) to block non-specific binding and then stained with fluorescence labeled antibodies (BD Biosciences, BioLegend or eBioscience). All cell suspensions were stained with a viability dye (Zombie Aqua) to exclude dead cells.

For specific analysis of DC, a lineage cocktail was used to exclude T, B, natural killer (NK) and erythroid cells in analysis of DC (anti-CD3 PE (145-2C11), anti-CD19 PE (1D3), anti-NK PE (2B4), anti-TER119 PE (TER-119). DC subpopulations were identified by using anti-I-A/I-E Alexa Fluor 488 (M5/114.15.2), anti-CD11c Alexa Fluor 700 (N418), anti-CD8α PE CY7 (53–6.7), anti-CD11b Violet Fluor 450 (M1/70), anti-CD103 biotin (2E7), anti-CD207 APC (eBioRMUL2), anti-CD5 Alexa Fluor 594 (53–7.3) and Streptavidin APC Cy7. Resident and migratory DC in SP and LN were analyzed as previously reported [38]. Lung cell suspensions were stained with the lineage cocktail (as before) and anti-Gr1 PerCP (RB6-8C) and anti-F4/80 APC (BM8) antibodies were used to discriminate macrophages. Also, lung DC were identified by using anti-I-A/I-E Alexa Fluor 488 (M5/114.15.2), anti-CD11c Alexa Fluor 700 (N418) and anti-CD5 Alexa Fluor 594 (53–7.3). For BMDC, we used anti-I-A/I-E Alexa Fluor 488 (M5/114.15.2), anti-B7.-1 PE CY5 (16-10A1), anti-CD11c PE CY7 (N418), anti-B7-2 APC (GL-1), anti-CD40 PE (3/23), anti-CD274 PerCP eFluor710 (MIH5) and anti-CD197 PE CF594 (4B12). The cells were acquired in an Accuri C6 Flow Cytometer (BD Biosciences) or in an Attune NxT Flow Cytometer (Thermo Fisher Scientific) and analyzed with Flowjo Software (Tree Star Inc).

### Cytokine production by DC

Purified CD11c+ SP DC were stimulated with LPS (1 μg/ml) and 24h later supernatants were harvested and IL-12 and IL-23 concentrations were detected by ELISA Ready-SET-Go kits (eBioscience, San Diego, CA).

### Real-time PCR

The expression of mRNA was quantified by Real-time PCR (RT-PCR) as described previously [42]. Briefly, total RNA was isolated from homogenized skin tissues by using TRIzol Reagent (GibcoBRL) according to the manufacturer´s instructions. Two micrograms of RNA were used for synthesis of cDNA with a cDNA synthesis kit from Bio-Rad Laboratories, Inc. (Hercules, CA). Real time PCR was performed with iQ SYBRO Green Supermix Kit (Bio-Rad Laboratories, Inc.) in Fast Real-Time PCR System QuantStudio6 Flex (Applied Biosystems) according to the manufacturer’s instructions. The expression level of cytokines was normalized to the house-keeping gene GAPDH in each sample and presented as relative level in data analysis.

The sequences for primers were as follows: IL-12p35, forward, 5’-CACAAGAACGAGAGTTGCC-3’, reverse, 5’-TCAAGTCCTCATAGATGCTACC-3’. IL-12p40, forward: 5’-ACGGCAGCAGAATAAATATGAG-3’, reverse, 5’-GGAGAAGTAGGAATGGGGAG-3’. IL-23p19, forward, 5’-AGATCTGAGAAGCAGGGAAC-3’, reverse, 5’-TGCCACTGCTGACTAGAAC-3’. IL-6, forward, 5’-GCCTTCCCTACTTCACAAGTCC-3’, reverses, 5’-TAGCCACTCCTTCTGTGACTCC-3’. IL-21, forward, 5’-TCATCATTGACCTCGTGGCCC-3’, reverse, 5’-ATCGTACTTCTCCACTTGCAATCCC-3’. TGF-β1, forward, 5’-ACCCTACTTCAGAATCGTCC-3’, reverse, 5’-ACAGTTCAATCCGCTGCTC-3’. IL-1β, forward, 5’-CAAATCTCACAGCAGCAC-3’, reverse, 5’-ACCGCTTTTCCATCTTCTTC-3’. IL-1α, forward, 5’-TCAGCACCTTACACCTACC-3’, reverse, 5’- GCAACTCCTTCAGCAACAC-3’. TNF-α: forward, 5’-GAGCACAGAAAGCATGATCC-3’, reverse, 5’-ACTTGGTGGTTTGCTACGAC-3’. IL-4, forward, 5’-ACGGATGCGACAAAAATCAC-3’, reverse, 5’- ACCTTGGAAGCCCTACAGAC-3’.

### Assessment of DC-induced T cell activation

To examine DC induced T cell proliferation, CD4+ T cells were purified from transgenic mice (2D2) and labeled with carboxyfluorescein succinimidyl ester (CFSE). SP DC were purified from WT or CD5^-/-^ mice by anti-CD11c antibody coupled MACS beads and cultured with CFSE-labeled 2D2 T cells in the presence of MOG_35-55_ peptide (2 μg/ml) and four days later, the percent of dividing cells were analyzed by flow cytometry. In further experiments, OTII or OTI T cells were purified using anti-CD4 or anti-CD8 MicroBeads, respectively (Miltenyi Biotec., Auburn, CA) and were labeled with CFSE [17, 18]. OT-II or OT-I CFSE+ T cells (2 x 10^6^ cells/ml) were incubated with BMDC (5 x 10^5^/ml) in 96 well culture plates in the presence of 2 μg/ml MHC class II peptide OVA_323–339_ (ISQAVHAAHAEINEAGR) or 1 μg/ml MHC class I peptide OVA_257–264_ (SIINFEKL) (AnaSpec, San Jose, CA), respectively [39]. Four days later, the percent of dividing cells were analyzed by flow cytometry.

To examine the ability of DC to stimulate T cells *in vivo*, CD5^-/-^ and WT BMDC were pulsed with the MHC class II or I peptides and injected subcutaneously in the footpad of WT mice (2 x 10^6^ cells/mouse). The mice were then injected intravenously with CFSE labeled OT-II or OT-I cells (6 x 10^6^ cells/mouse). The draining popliteal LN of the mice were harvested 4 days later and CFSE positive cells were analyzed for cell division by flow cytometry.

### Examination of cytokine production by T cells

OT-II CD4+ T cells were purified and incubated with purified SP CD11c+ DC or BMDC in the presence of 2 μg/ml MHC class II peptide as described above. T cell cultures without addition of any peptide served as controls. Supernatants were collected 4 days after cultures. Concentrations of IL-2, IL-17 and IFN-γ in supernatants were measured by ELISA as described [38].

To examine cytokine producing cells, T cells were harvested 4 days after the cultures and stimulated with PMA and ionomycin in the presence of a Golgi blocker for 4 hours as described [38]. Briefly, CD4+ T cells were gated and IL-2, IL-4, IL-17 and IFN-γ producing cells were analyzed by flow cytometry. Cytokine production by hapten-primed T cells was examined after isolation of primed T cells from the draining LN of mice that were sensitized with DNBS labeled WT or CD5^-/-^ SP DC. BMDC were labeled with DNBS and cultured with primed or control naïve T cells (2 x 10^6^ T cells/ml and 2 x 10^5^ DC/ml). Supernatants were harvested 48 hours after cultures and IL-2, IL-17 and IFN-γ were measured by ELISA as described [38].

### Induction of anti-tumor immune responses by OVA-pulsed DC

WT and CD5^-/-^ BMDC (1 x 10^6^ cells/ml) were pulsed with 100 μg/ml OVA as described in our previous studies [39]. Mice were injected subcutaneously with OVA pulsed WT or CD5^-/-^BMDC (2 x 10^6^ cells/mouse in 200 μl PBS). Seven days after the immunization, the mice were inoculated subcutaneously with mouse E.G7 tumor cells (4 x 10^6^ cells/mouse). Tumor growth was monitored every 3 days in a double-blinded way. Tumor sizes were calculated with the formula: Tumor Size = L × S × H × π/6 (L: long diameter, S: short diameter, H: height).

### Contact hypersensitivity responses

Induction and measurement of Contact Hypersensitivity (CHS) responses were described previously [38]. In the current studies, BMDC or purified SP DC were labeled with 5mM of Di-nitrobenzene Sulfonic Acid (DNBS, Sigma St. Louis, MO) at 37°C for 15 minutes. For sensitization of mice, hapten-labeled DC were injected subcutaneously in WT mice (1 x 10^6^ DC/mouse). Five days after the sensitization, the mice were challenged with 0.2% DNFB on ear skin. Ear swelling was read 24 hours after the challenge in a double-blinded way. Mice which were not sensitized but challenged served as negative controls.

### Statistical analysis

All data are presented as mean ± Standard error (SEM). For all experiments the two-tailed Student’s *t*-test was applied for statistical analysis with p< 0.05 being considered statistically significant.

## Results

### CD5 is expressed by CD11c+ DC in lymphoid and non-lymphoid tissues

**Fig 1:**
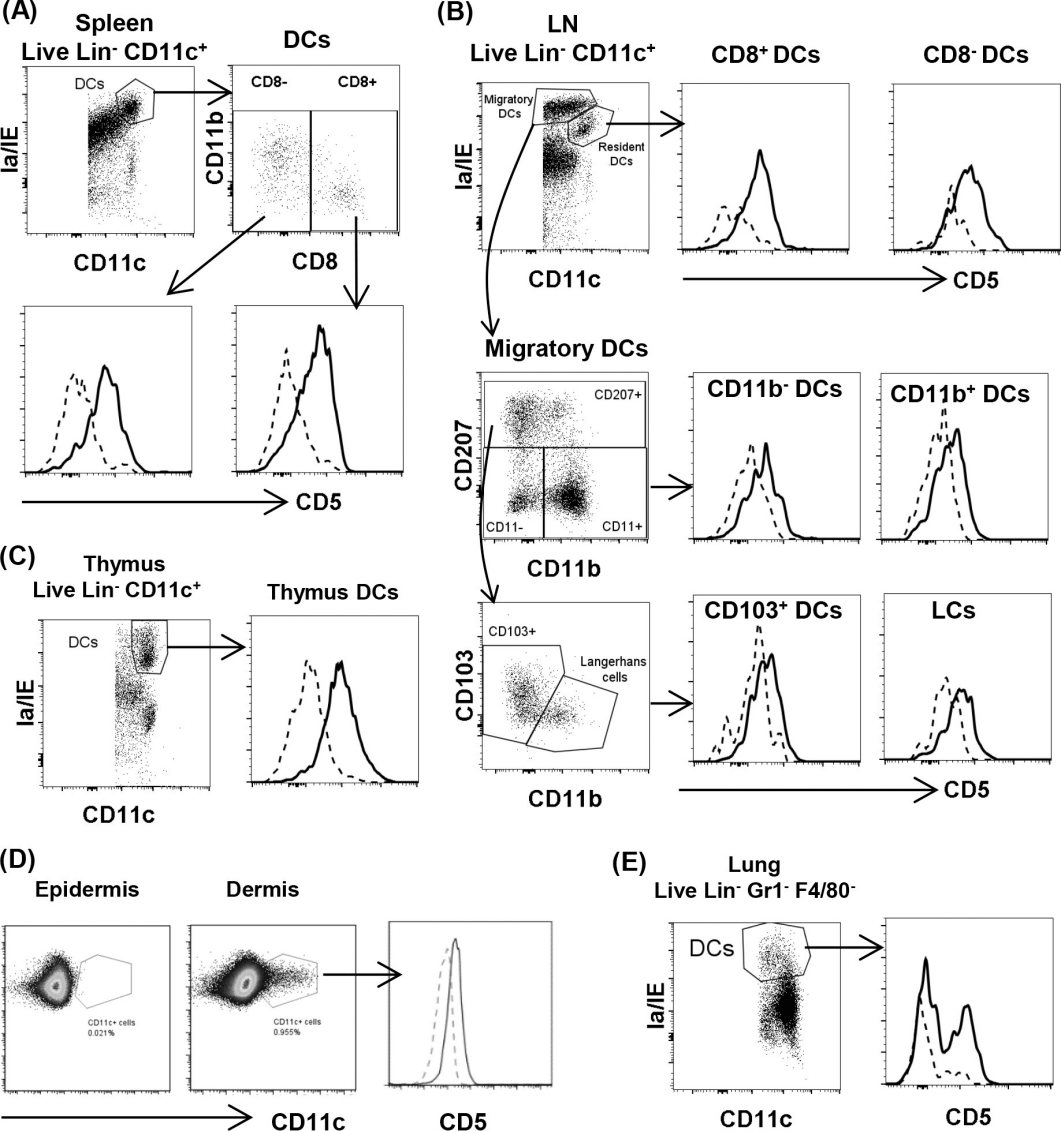
Expression of CD5 on dendritic cells. DC from various lymphoid and non-lymphoid organs are analyzed. In histograms, CD5 staining (solid line) is compared to fluorescence minus one (FMO) control (dashed line). (A) Gated SP DC (Lin-/CD11c+/Ia-IE+) express CD5. Both CD8α+ and CD8α- subsets express a similar level of CD5. (B) Resident and migratory DC from LN are gated and analyzed. CD8α+ and CD8α- resident DC express a higher level of CD5 than migratory DC which include four subpopulations (CD11b+, CD11b-, CD103+ and Langerhans CD207+/CD103-). (C) Gated thymus DC (Lin-/CD11c+/Ia-IE+) express CD5. (D) Dermal CD11c+ DC express CD5. Only few CD11c+ cells from the epidermal cells can be detected. (E) DC from the lung (Lin-/Gr1-/F4/80-/CD11c+/Ia/IE+) express CD5. The data are representative of 2–3 independent experiments.

We interrogated if CD5 was expressed on DC from SP, LN, thymus, lungs and skin from naïve C57BL/6 and Balb/c mice. Results showed that live Lin-/CD11c+/Ia/IE+ SP DC expressed CD5 with similar levels on CD8α+ and CD8α- subsets (Fig 1A). Similarly, CD8α+ and CD8α- resident DC (Ia/IE^low^/CD11c^hi^) from lymph nodes expressed CD5. Migratory DC (Ia/IE^hi^/CD11c^low^) in lymph nodes, including Langerhans cells (CD207+/CD103-) and dermal DC subpopulations (CD11b+ DC, CD11b-DC and CD103+ DC) expressed detectable levels of CD5 which were lower than those of resident DC (Fig 1B). CD5 expression was also evident on thymic DC (Fig 1C). CD11c+ DC in non-lymphoid organs such as dermis expressed low levels of CD5 (Fig 1D). The epidermis contained too few DC to confidently detect CD5 expression (Fig 1D). In the lungs, a proportion of the Lin-/Gr-1-/F480- DC (CD11c+ gated) expressed CD5 (Fig 1E). Collectively, the data indicate that CD5 is broadly expressed on DC in murine lymphoid and non-lymphoid tissues. Notably, migratory DC in LN represent a population derived from the skin and they express a lower level of CD5 than resident DC.

### Outcome of CD5 deficiency in DC

**Fig 2:**
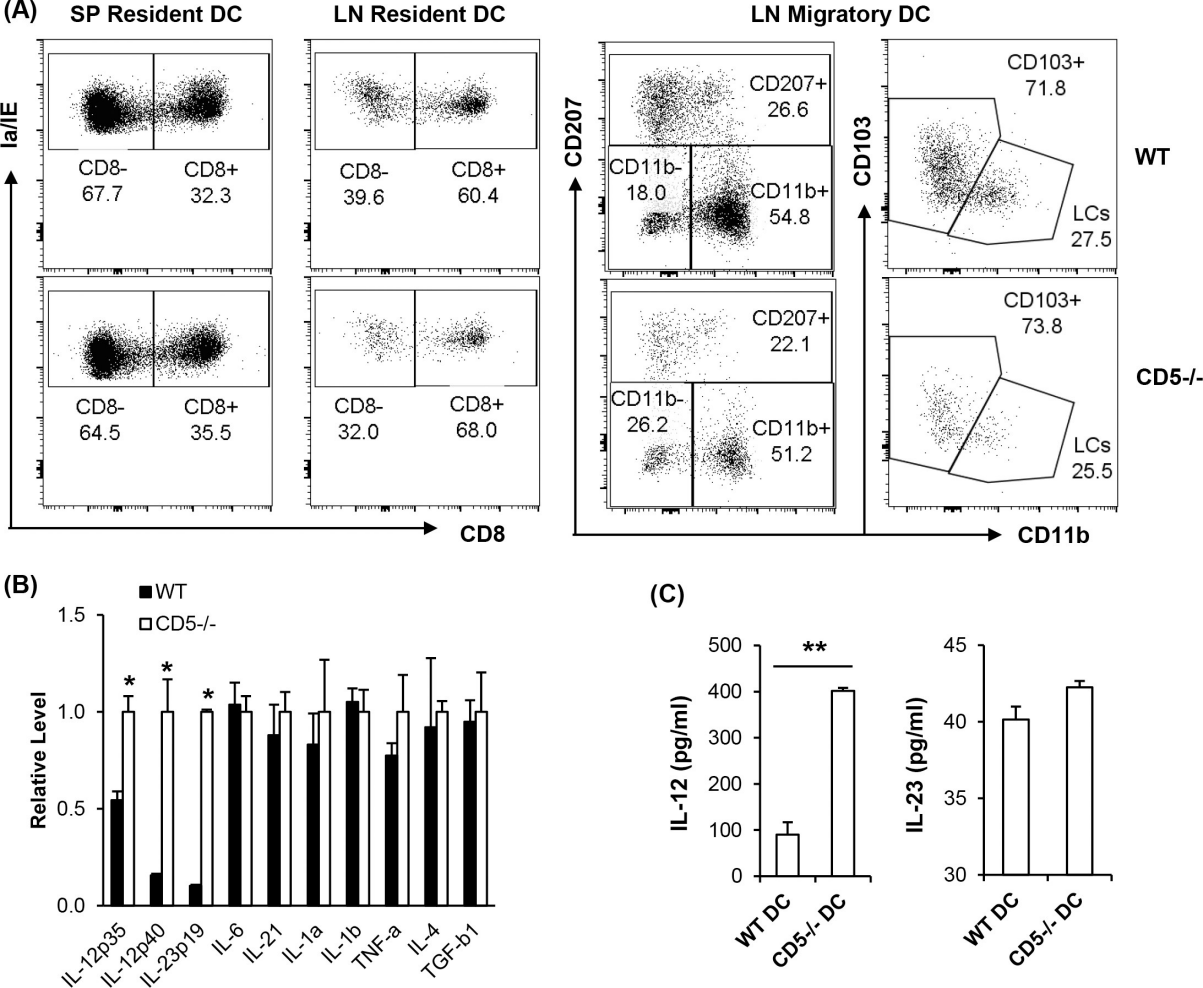
Effects of CD5 deficiency on DC. (A) DC from wild-type (WT) (upper panels) and CD5^-/-^ (bottom panels) mice are gated as described in the Fig 1 and resident and migratory DC populations are analyzed. The numbers in the graphs show the percentage of the indicated DC subsets. (B) CD5 deficiency alters cytokine production by BMDC. Levels of cytokine mRNA from WT and CD5^-/-^ BMDC are quantified by real time RT-PCR (n = 4, * P<0.05). (C) CD5 deficiency increases IL-12 production by DC. SP DC from WT and CD5^-/-^ were purified by CD11c coupled MACS beads and stimulated with LPS for 24 hours. Cytokine concentrations in culture supernatants were measured by ELISA (n = 3, **P<0.01). The data are representative of 2–3 independent experiments (two-tailed Student’s *t*-test).

We interrogated if development of DC was affected in CD5-/- mice. We confirmed that CD5 was not expressed in CD5^-/-^ DC by assaying for CD5 mRNA and surface protein expression (S1 Fig). Results showed that CD5 deficiency did not alter the overall composition of DC as well as resident and migratory DC subpopulations in SP and LN neither in percentages (Fig 2A and S2 Fig) nor in total cell numbers (S3 Fig). There was no significant difference between WT and CD5^-/-^ mice in the expression levels of CD40, Ia/IE, B7.1, B7.2, B7-H1 and B7-DC on SP DC (S1 Fig). We also did not find a significant change in the number and percentage of DC in lymphoid organs between WT and CD5^-/-^ mice, indicating that CD5 is not involved in DC development at homeostasis.

To determine if CD5 had an effect on the function of DC, BMDC generated from WT and CD5^-/-^ mice were stimulated with LPS for 24h and analyzed for expression levels of class II, costimulatory molecules (CD40, B7-1, B7-2), co-inhibitory molecules (B7-H1, B7-DC) and cytokines secreted by DC important for activation and differentiation of T cells. We observed no significant differences in expression of any cell surface molecule between CD5^-/-^ and WT BMDC (S1 Fig). CD5^-/-^ BMDC expressed significantly higher levels of IL-12p40, IL-12p35 and IL-23p19 mRNA than WT BMDC (Fig 2B). The expression levels of IL-6, IL-21, IL-1β, IL-1α, TNF-α, IL-4 and TGF-β1 mRNA was equivalent between CD5^-/-^ and WT BMDC (Fig 2B). To determine if BMDC data reflects primary DC, we stimulated purified DC from spleens of WT and CD5^-/-^ mice with LPS for 24 hours and cytokine production was measured by ELISA. Results showed that CD5^-/-^ SP DC produced significantly higher levels of IL-12 but not IL-23 than WT counterparts (Fig 2C). The expression of B7-1 and B7-2 on CD5^-/-^ SP DC was not significantly different from that on WT SP DC (S1 Fig). Overall, these results suggest that CD5 regulates IL-12 production with no significant effect on DC development and expansion.

### CD5 deficiency in DC enhances their activity to stimulate T cells and induce immune responses

**Fig 3:**
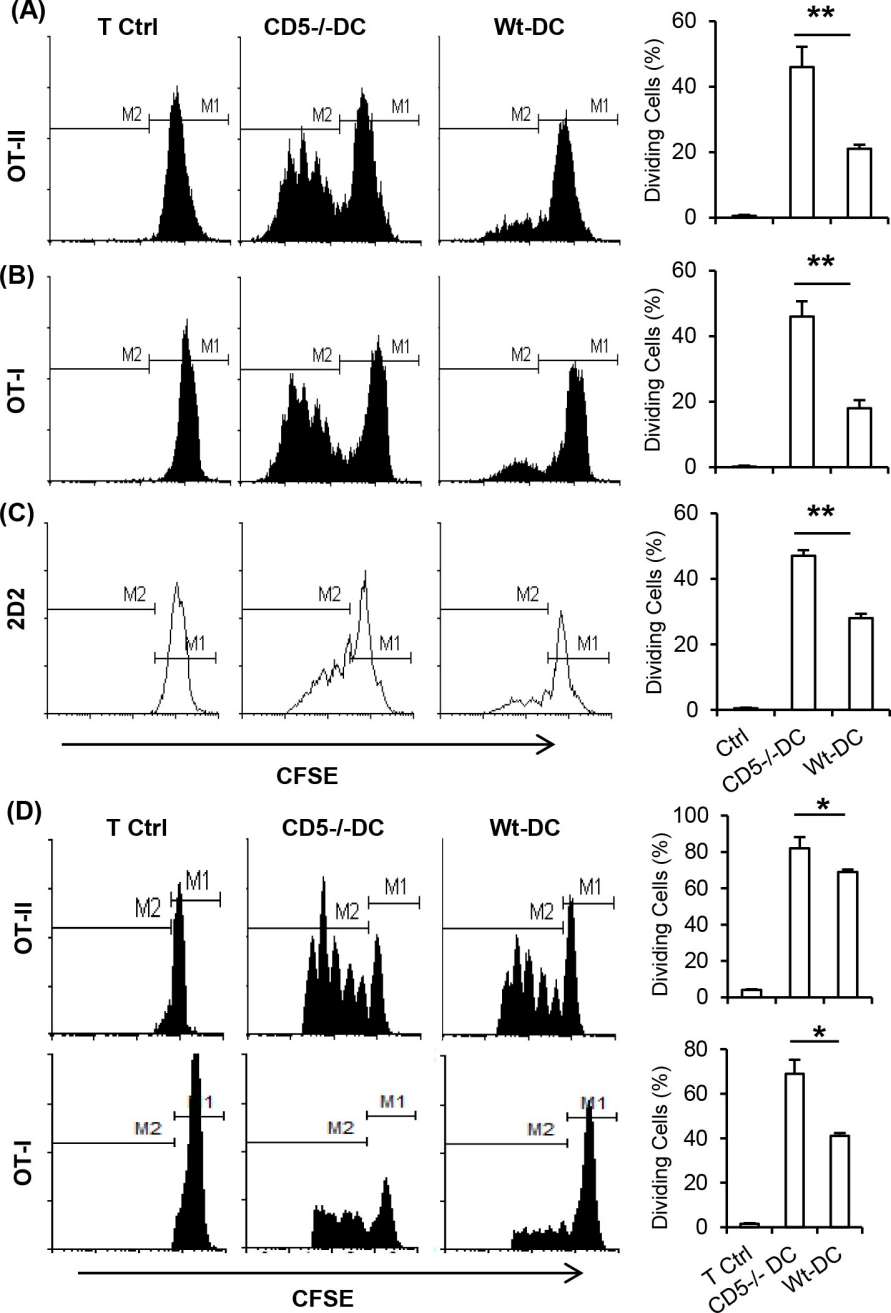
CD5 deficiency enhances the capacity of DC to activate CD4+ and CD8+ T cells. OT-II (A) or OT-I (B) cells were cultured with OVA peptides pulsed BMDC. The cultures without OVA peptides served as controls (T Ctrl) (n = 5). (C) CD4+ T cells from 2D2 mice were stimulated with CD11c+ SP DC from WT or CD5^-/-^ mice in the presence of MOG_35-55_ peptide (n = 5). (D) Naïve WT mice were injected subcutaneously with OVA peptides pulsed BMDC into rear footpads followed by intravenous injection of CFSE labeled OT-II or OT-I cells. Control mice received CFSE labeled T cells but no DC (Ctrl). The draining popliteal LN were analyzed (n = 6). The data are representative of 3–5 independent experiments (two-tailed Student’s *t*-test, *P<0.05, **P<0.01).

CD5 in T cells regulates T cell activation [4, 7, 9]and DC as antigen presenting cells have an essential function for the proliferation of T cells and induction of immune responses [20–23]. However, it has not been investigated whether CD5 expressed on murine DC has a role in modulating T cell activation. To address this question, BMDC generated from WT and CD5^-/-^ mice were pulsed with MHC class II or class I specific OVA peptides and co-cultured with OT-II or OT-I T cells. Results showed that CD5^-/-^ BMDC significantly promoted higher level of OT-II and OT-I T cell proliferation than WT BMDC (Fig 3A and 3B). We also found that purified SP DC from CD5^-/-^ mice induced a significantly higher level of MOG_35-55_ peptide-specific 2D2 CD4+ T cell proliferation compared to WT SP DC (**Fig 3C)**.

To determine if CD5 modulates DC-induced activation of CD4+ and CD8+ T cells *in vivo*, naïve WT mice were injected subcutaneously with OVA peptide pulsed WT or CD5^-/-^ BMDC into the rear footpads, followed by intravenous injection with CFSE labeled OT-II or OT-I cells. The draining popliteal LN were harvested and the proliferation of CFSE-labeled T cells was analyzed by flow cytometry. Results showed that mice injected with CD5^-/-^ BMDC induced a significantly higher level of OT-I and OT-II T cell proliferation than those injected with WT BMDC, a result recapitulating *in vitro* cultures (Fig 3D).

**Fig 4:**
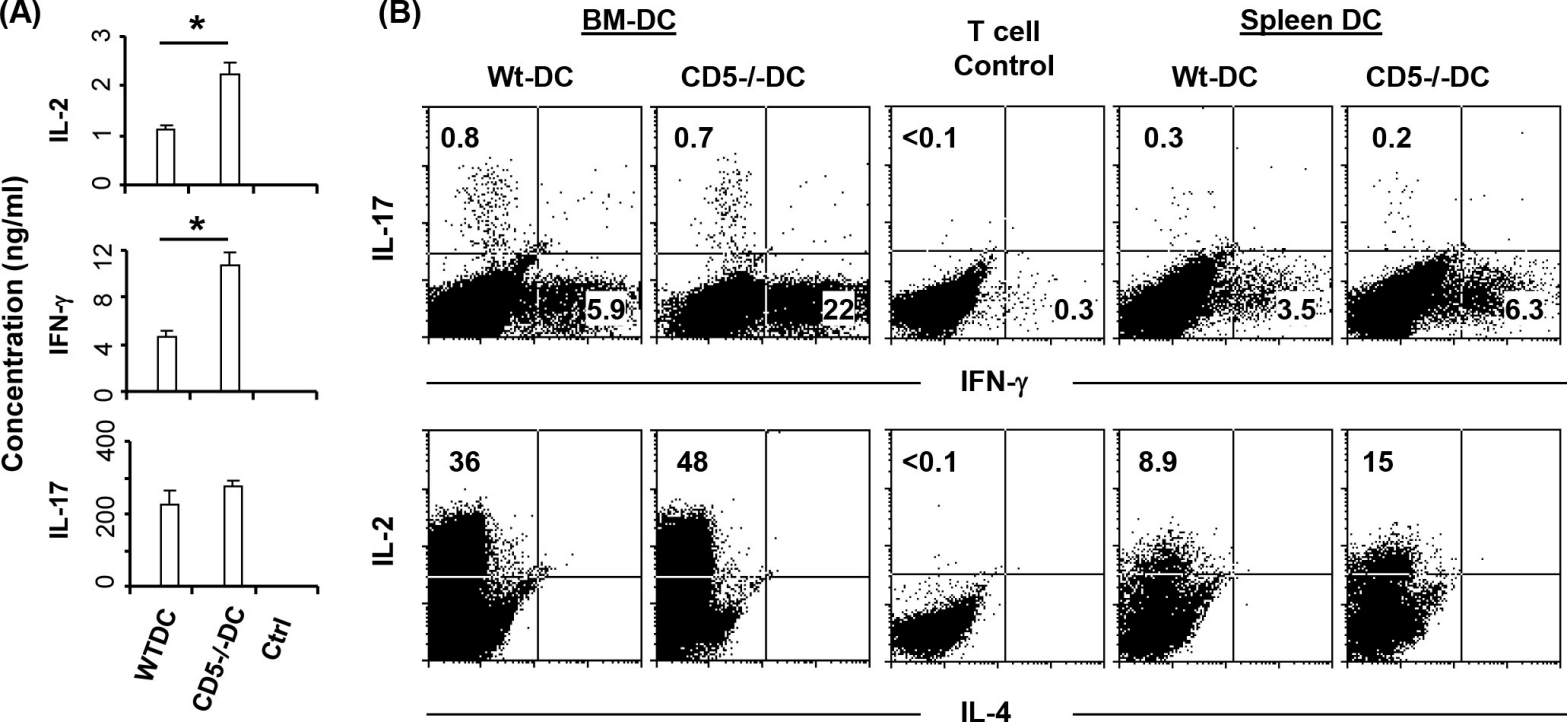
CD5 deficiency enhances the ability of DC to stimulate the production of Th1 cytokines by T cells. (A) BMDC were cultured with OT-II cells for 4 days in the presence of the OVA peptides. Control T cells (Ctrl) were cultured with BMDC which were not pulsed with OVA. Supernatants from T cell/DC cultures were harvested and cytokine concentrations were measured by ELISA (n = 4, *P<0.05). UD: undetected. (B) Purified CD11c+ SP DC or BMDC were pulsed with OVA peptides and cultured with OT-II cells for 4 days. The percent of cytokine producing cells were detected by intracellular cytokine staining and analyzed by flow cytometry. The data are representative of 2–3 independent experiments (two-tailed Student’s *t*-test).

Our results show that CD5 deficiency significantly upregulates the production of IL-12 by BMDC and SP DC compared to WT DC (Fig 2B and 2C), a cytokine necessary for the differentiation of naïve CD4+ T cells into Th1 cells [43–45]. We therefore examined if CD5 in DC has a role in the differentiation of CD4+ Th1 and Th17 helper cells by co-culturing OT-II cells with OVA peptide-pulsed WT or CD5^-/-^ DC and the lineage specific cytokine production was measured by ELISA. Results showed that CD5^-/-^ DC induced significantly higher levels of Th1 cytokine IFN-γ and IL-2 than WT DC (Fig 4A), whereas IL-17 production was not significantly altered. Further experiments with intracellular cytokine staining supported an increase in IL-2 and IFN-γ, but not IL-4 or IL-17, producing T cells in the cultures with either BMDC or SP DC from CD5^-/-^ mice compared to those from WT mice (Fig 4B).

### CD5 deficiency in DC enhances anti-tumor immunity

**Fig 5:**
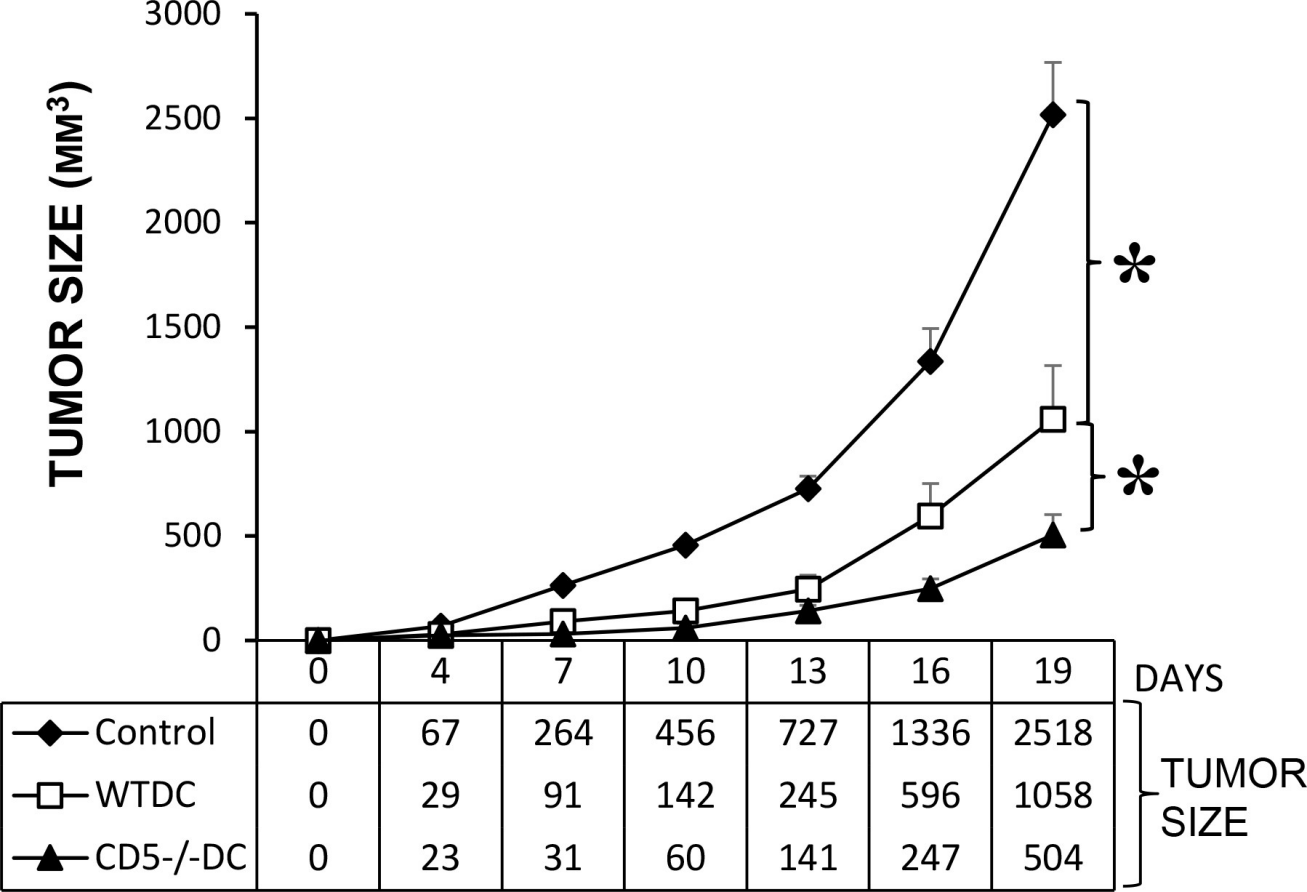
CD5^-/-^ DC are more potent than WT DC to induce anti-tumor immunity in animals. WT mice were immunized subcutaneously with OVA pulsed BMDC generated from WT or CD5^-/-^ mice. Control mice were not immunized. The mice were then inoculated subcutaneously with mouse EG.7 tumor cells. Tumor size was measured every 3 days (n = 5, *P<0.05). The data are presented as mean ± SEM and are representative of 2 independent experiments (two-tailed Student’s *t*-test).

CD4+ Th1 and CD8+ T cells have major roles in anti-tumor immunity [46, 47]. To examine whether the enhanced ability of CD5^-/-^ DC to promote CD4+ Th1 and CD8+ T cells leads to more robust anti-tumor immune responses, naϊve mice were immunized subcutaneously with WT or CD5^-/-^ BMDC pulsed with OVA. Controls received no BMDC. The mice were then inoculated with EG.7 tumor cells (EL4 cells transfected with OVA gene). Results show tumor size in mice immunized with OVA pulsed WT or CD5^-/-^ BMDC were significantly lower than controls; importantly, CD5^-/-^DC were more potent than WT DC in the induction of anti-tumor immunity (Fig 5). The tumor size in CD5^-/-^ BMDC immunized mice was less than the half of that in WT DC immunized mice at the indicated times of measurements. The results support a regulatory role for CD5 in DC in the context of proliferation of CD4+ and CD8+ T cells and demonstrate that blockade of CD5 signals in DC enhances anti-tumor immune responses.

### CD5 regulates contact hypersensitivity (CHS) responses

**Fig 6:**
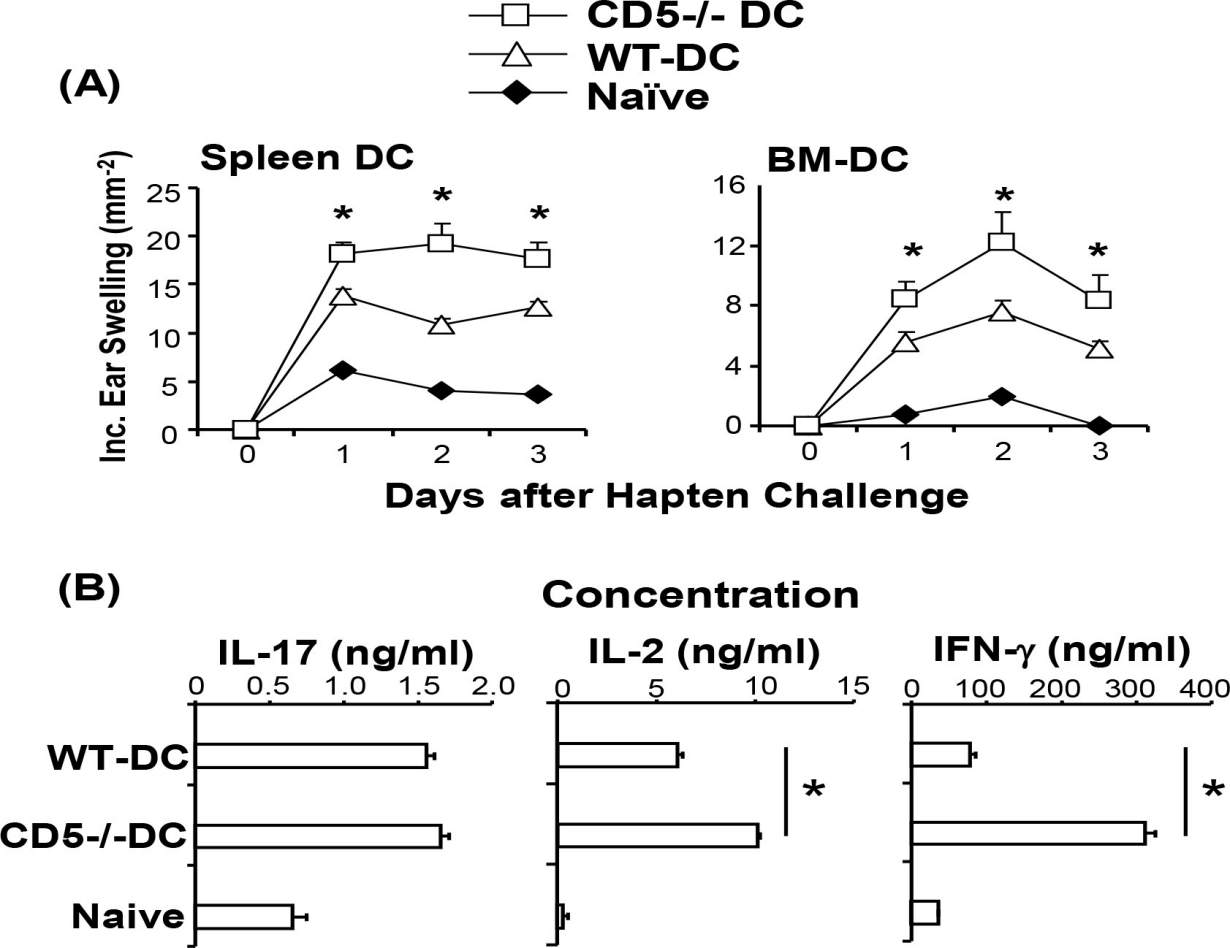
CD5^-/-^ DC enhance contact hypersensitivity responses (CHS). SP DC or BMDC from WT (WT-DC) or CD5^-/-^ (CD5^-/-^DC) mice were pulsed with DNBS and injected subcutaneously in naïve mice. (A). Sensitized mice were challenged with DNFB and ear swelling was measured. Naïve mice which were not injected with DC but challenged with DNFB served as controls (n = 5, * P<0.05). (B) T cells from the draining LN of the immunized mice were stimulated with DNBS-pulsed WT BMDC. Cytokine concentrations were measured by ELISA. T cells from naïve mice served as controls (n = 4, * P<0.05). The data presented are mean ± SEM and are representative of 2–3 independent experiments (two-tailed Student’s *t*-test).

Our results reveal a specific role for CD5 in DC in regulating CD4 and CD8 proliferation and differentiation *in vitro* and *in vivo*. To further address this biology, we examined if CD5 on DC regulated the contact hypersensitivity (CHS) response which is a delayed type hypersensitivity mediated by Th1 and CD8+ T cells and requires IFN-γ [42, 48–52]. We sensitized naïve mice by a subcutaneous injection with purified WT and CD5^-/-^ SP DC labeled with hapten DNBS. The sensitized mice were then challenged with DNFB and CHS responses were measured. Results showed that CHS responses was significantly greater in mice sensitized with hapten-labeled SP DC from CD5^-/-^ mice than that with WT DC (Fig 6A, left panel). Similarly, hapten-labeled BMDC from CD5^-/-^ mice induced significantly greater CHS than WT BMDC (Fig 6A, right panel). T cells were isolated from the draining LN of mice sensitized with hapten-labeled WT or CD5^-/-^ SP DC and re-stimulated with hapten-labeled WT BMDC. Results showed that T cells from mice which were sensitized with CD5^-/-^ DC produced significantly higher levels of IFN-γ and IL-2, but not IL-17, than those from mice sensitized with WT DC (Fig 6B). This cytokine expression pattern recapitulates that of T cells stimulated with OVA pulsed WT or CD5^-/-^ DC (Fig 4A and 4B).

### Re-expression of CD5 in CD5^-/-^ DC restores their ability to activate T cells

**Fig 7:**
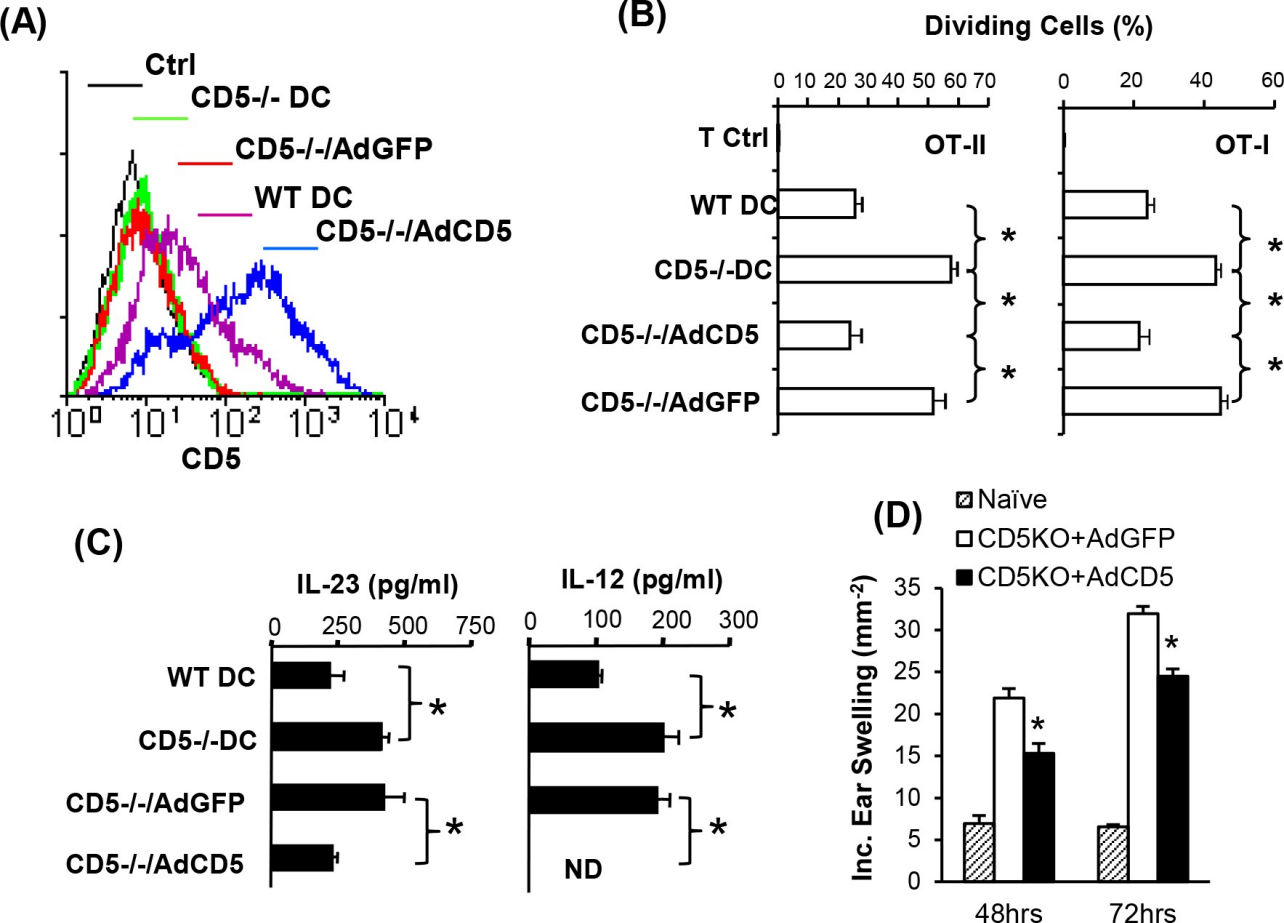
Re-expression of CD5 restores regulatory activity to CD5^-/-^ DC. (A). Restoration of CD5 in CD5^-/-^ BMDC transfected with Ad-GFP (CD5^-/-^ /AdGFP) or Ad-CD5 (CD5^-/-^ /AdCD5). Ctrl: Isotype-matched IgG control. (B). The restoration of CD5 diminishes the activity of CD5^-/-^ DC to stimulate proliferation of CD4+ and CD8+ T cells. T cell control (T Ctrl) was cultured without DC (n = 4). (C) The re-expression of CD5 expression inhibits cytokine production by CD5^-/-^ DC (n = 4). Note that IL-12 was below the detectable level (UD) in the CD5^-/-^/AdCD5 group. (D) The re-expression of CD5 expression attenuates the ability of CD5^-/-^ DC to induce CHS responses (n = 6). The data are presented as mean ± SEM and are representative of 2–3 independent experiments (two-tailed Student’s *t*-test, *P<0.05, ** P<0.01).

In the CD5^-/-^ mice, CD5 is absent from the onset of development. To exclude the possibility that our observations reflect an unrecognized developmental change in DC, we restored CD5 expression in CD5^-/-^ BMDC using adenovirus encoding CD5 (Ad-CD5) or GFP (Ad-GFP). Infection with Ad-CD5 successfully restored the expression of CD5 in CD5^-/-^ BMDC to levels higher than endogenous CD5 on WT BMDC (Fig 7A). The ability of BMDC to regulate T cell responses were comparable to CD11c+ DC from SP, although they expressed lower levels of CD5 (Fig 1). Control Ad-GFP infected BMDC did not express any CD5 (Fig 7A). Ad-CD5 or Ad-GFP CD5^-/-^ infected BMDC were pulsed with OVA and cultured with OT-II or OT-I cells. Results show that Ad-CD5 infected CD5^-/-^ BMDC induced proliferation of OT-II or OT-I T cells to levels equivalent to that by WT BMDC (Fig 7B). Ad-GFP infection had no effect; its ability to induce proliferation was as efficient as uninfected CD5^-/-^ BMDC and higher than WT BMDC (Fig 7B). CD5^-/-^ BM DC produced significantly higher levels of IL-12 and IL-23 than WT BMDC (Fig 7C). This is in accordance with the difference in the mRNA level of these cytokines in WT and CD5^-/-^ BMDC (Fig 2B). Restoration of CD5 expression in CD5^-/-^ BMDC significantly reduced the production of IL-12 and IL-23. Infection of CD5^-/-^ BMDC with Ad-GFP did not have a significant effect on cytokine production compared to control CD5^-/-^ BMDC (Fig 7C). To determine the effect of CD5 restoration on the function of DC in the induction of immune responses, Ad-CD5 and Ad-GFP infected DC were labeled with hapten DNBS and injected subcutaneously to sensitize naïve mice. We found that Ad-CD5 transfected CD5^-/-^ BMDC induced a significant lower level of CHS responses than Ad-GFP transfected CD5^-/-^DC (Fig 7D). Collectively, the results indicate that the restoration of CD5 expression diminishes the activity of CD5^-/-^ DC to stimulate T cell proliferation, produce IL-12 and IL-23 and to induce immune responses.

## Discussion

The role of CD5 in the regulation of T cell activation and tolerance has been extensively studied [7–9]. Our finding has revealed a previously unrecognized function for CD5 in regulation of DC functions and added new insights into CD5 mediated effects on immune responses.

It was not well examined whether murine DC are able to express CD5, although a few reports show that human and rat DC express CD5 mRNA [31, 32]. DC from human tonsil and lymph nodes express CD5 and blood cDC2 (HLA-DR+/CD11+/CD1c+) have CD5hi and CD5low subsets [33]. Subpopulations of human blood pDC, epidermal LC (CD1a^hi^/Langerin+) and dermal DC (HLA-DR+/CD1a^dim^) also express CD5 [34, 35]. The results from this study demonstrate that the expression of CD5 is a common feature of murine DC. We show that CD5 is expressed by Lin-/CD11c+/Ia/IE+ DC in lymphoid organs, dermis and lungs. CD8α+ and CD8α- DC subsets (Ia/IE^low^/CD11c^hi^) from SP and LN express a higher level of CD5 than migratory LN DC (Ia/IE^hi^/CD11c^low^) represented by Langerhans cells (CD207+/CD103-) and dermal DC (CD11b+ DC, CD11b-DC and CD103+ DC). The lower expression of CD5 on DC from peripheral non-lymphoid organs supports the concept that tissue resident DC are CD5^lo^. These findings were common to both C57BL/6 and Balb/c mice, indicating that it is not restricted to certain mouse strains. Human DC generated from human CD34+ hematopoietic progenitors in cultures express a low level of CD5 [35]. Similarly, murine BMDC in this study, a population that may include a small proportion of monocyte-derived macrophages [53], expressed a low level of CD5.

Collectively, our study and reports from human studies demonstrate that DC from lymphoid organs and non-lymphoid organs express CD5 although the expression pattern is not identical.

CD5 is not necessary for the development DC subpopulations *in vivo* or generation of BMDC. However, CD5 does regulate the production of IL-12 in BMDC and SP DC (Figs 2B, 2C and 7C). Literature well demonstrates that IL-12 promotes T cell proliferation [54, 55] and is necessary for differentiation of naïve CD4 T cells to Th1 cells which produce IL-2 and IFN-γ [43–45]. We show that CD5^-/-^ DC induce significantly higher levels of Th1 cell differentiation than WT DC. Moreover, CD5^-/-^ DC promote greater CD4 and CD8 T cell proliferation than WT DC *in vitro* and *in vivo*, probably attributable to differences in IL-12 secretion. The transfer of peptide pulsed CD5^-/-^ DC induces a significantly higher proliferation of T cells in LN than WT DC. It is not excluded that the transfer of peptide from migratory DC to lymph node resident DC in the recipient mice might occur in the experiments as reported [56, 57]. However, it may not contradict the increased activity of CD5^-/-^ DC since the recipient mice were wild type mice and wild type DC were less potent than CD5^-/-^ DC to induce proliferation of T cells. The increase in IL-23 production by CD5^-/-^ BMDC but not by CD5^-/-^ SP DC compared to their WT counterparts probably represents differences between *in vitro* and *in vivo* DC. However, both BMDC and SP DC from CD5^-/-^ mice induce a higher level of Th1 cells than their WT counterparts *in vitro* and *in vivo*, indicating that the increase of IL-12 by CD5^-/-^ DC may be a key mechanism for the enhancement of T cell activation and Th1 cell development. Taken together, the regulation of IL-12 has a key role in CD5 mediated effects on DC activity to activate T cells and to induce immune responses.

In human, CD5 expression levels on DC correlates directly with ability to stimulate T cell proliferation which is opposite to our findings in mice [33–35]. Multiple-factors can explain this apparent discordance with our findings with DC from CD5^-/-^ mice. These include the role of DC in allogeneic T cell responses (human studies) vs TCR-dependent T cell responses (this study) and the possibility that CD5+ and CD5- or CD5^low^ DC may represent two different DC subsets which also differ in other features such as maturation and activation (human studies). In fact, human blood CD5^low^ DC induce a higher level of IFN-γ+ T cells [33] whereas human skin CD5- DC induce a lower level of IFN-γ+ T cells [35] than their CD5+ counterparts. However, in both studies, CD5+ DC induce a higher level of T cell proliferation than CD5- or CD5^low^ DC. Nevertheless, mechanisms for the increased IFN-γ producing T cells induced by CD5^low^ or CD5- DC (human studies) remain to be elucidated. In our studies, we show that knockout of CD5 enhances the activity of DC to stimulate T cell proliferation, and importantly, to induce immune responses in two animal models. Moreover, we show that CD5 deficiency in DC increases IL-12 production and consequently enhances Th1 cell development. Importantly, these effects are reversed by restoration of CD5 expression in CD5^-/-^ DC. Interestingly, over expression of CD5 in the CD5^-/-^ DC resulted in undetectable levels of IL-12, implicating that production of this cytokine is regulated by CD5 expression levels (Fig 7C). This observation reflects the biologically relevance of CD5 expression levels in some immunological settings previously reported in literature [58]. Altogether, the composite data indicate that the ability of DC to stimulate T cell responses depends not only on CD5 expression levels but also on the nature of specific DC subsets.

Th1 and CD8+ T cells play a central role in immune responses against tumors [46, 47]. DC based immune therapy that enhance Th1 and CD8+ T cell immune responses has been successfully applied in treatment of cancer patients [59, 60]. However, improvement in efficiency of this approach is highly desired. Our data demonstrate that CD5^-/-^ DC are more effective than WT DC in the induction of immune responses in tumor and CHS models indicating a regulatory role for CD5 in DC. This gain in activity is directly attributable to the absence of CD5 since reconstitution of CD5 expression in DC restored CHS responses to that of WT DC. Our study presents a novel strategy where blockade of CD5 in DC to increase the production of IL-12 and enhance Th1 and CD8+ T cell responses can be harnessed for immune therapy against tumors.

The molecular mechanism underlying CD5-dependent regulation of DC function observed in this study needs further investigations. Domains within CD5 cytoplasmic tail previously shown to be important in regulation of T cell activation and differentiation such as CK2 binding and ITIM-like domains may have similar function in DC. Homotypic CD5-CD5 interactions, as suggested in previous studies in T cells [61], may also explain how DC regulates T cell proliferation. However, the regulation of IL-12 production is specific for CD5 mediated effects in DC.

## Conclusions

In summary, the current study demonstrates that CD5 regulates the activity of DC to induce T cell proliferation and immune responses. CD5 deficiency enhances the ability of DC to activate CD4+ and CD8+ T cells and promote Th1 cell differentiation. CD5^-/-^ DC are more potent than WT DC in the induction of anti-tumor immunity and CHS responses. CD5 has an inhibitory effect on IL-12 production by DC. Our study reveals a previously unknown biology of CD5 on DC activity and elucidates a novel mechanism for DC mediated immune responses in inflammatory diseases.

## Supporting information

S1 Fig Effects of CD5 deficiency on DC phenotype. (TIF)

S2 Fig CD5 deficiency does not have a significant effect on DC composition. (TIF)

S3 Fig CD5 deficiency does not have a significant effect on the total numbers of DC population. (TIF)
