# Supplementary figures and images for: CD5 on dendritic cells regulates CD4+ and CD8+ T cell activation and induction of immune responses
Source: PLoS One. 2019 Sep 6;14(9):e0222301. doi: 10.1371/journal.pone.0222301 (PMC6730919; doi:10.1371/journal.pone.0222301)

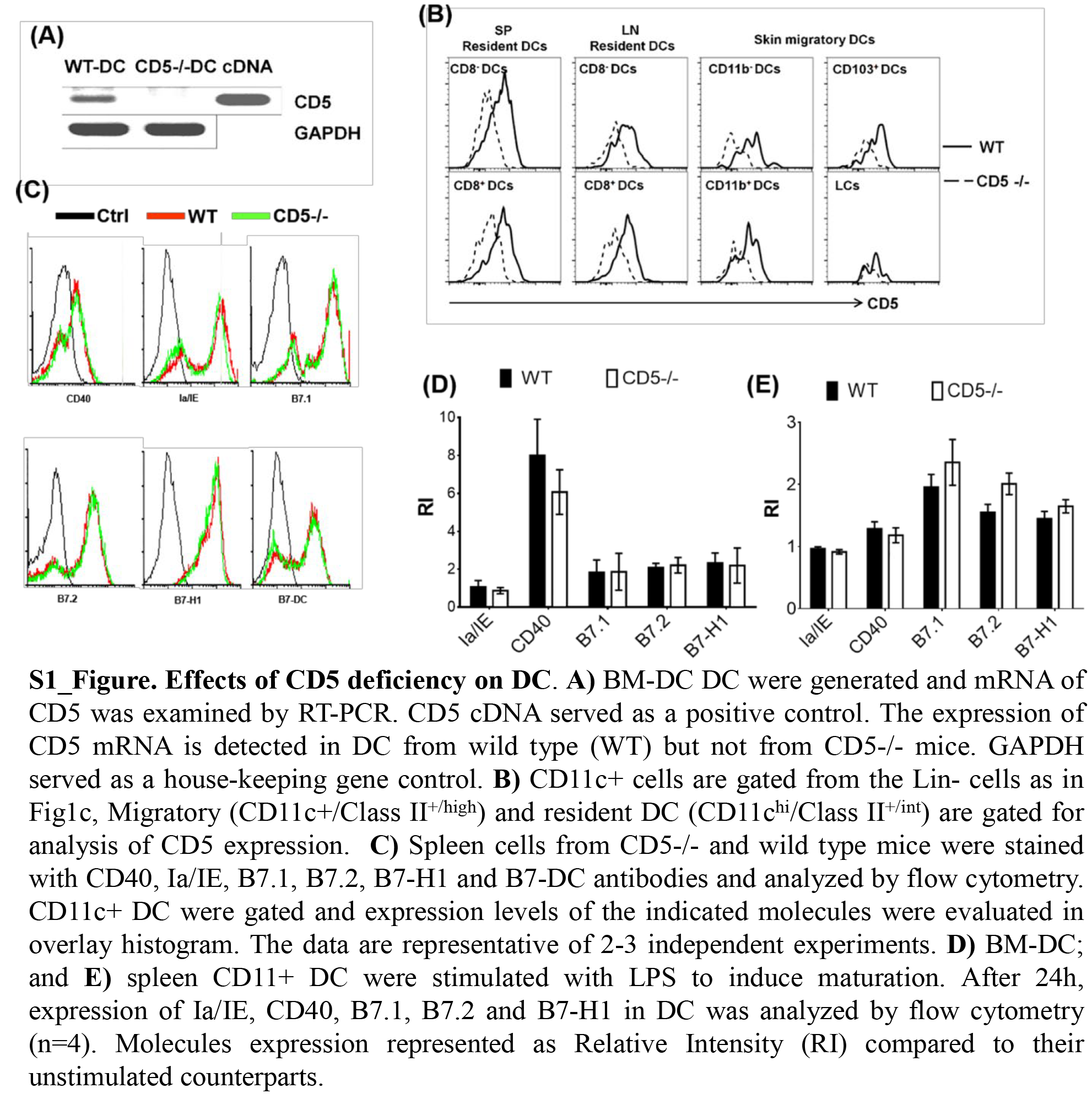

Supplement: S1 Fig — (TIF) [file pone.0222301.s001.tif]

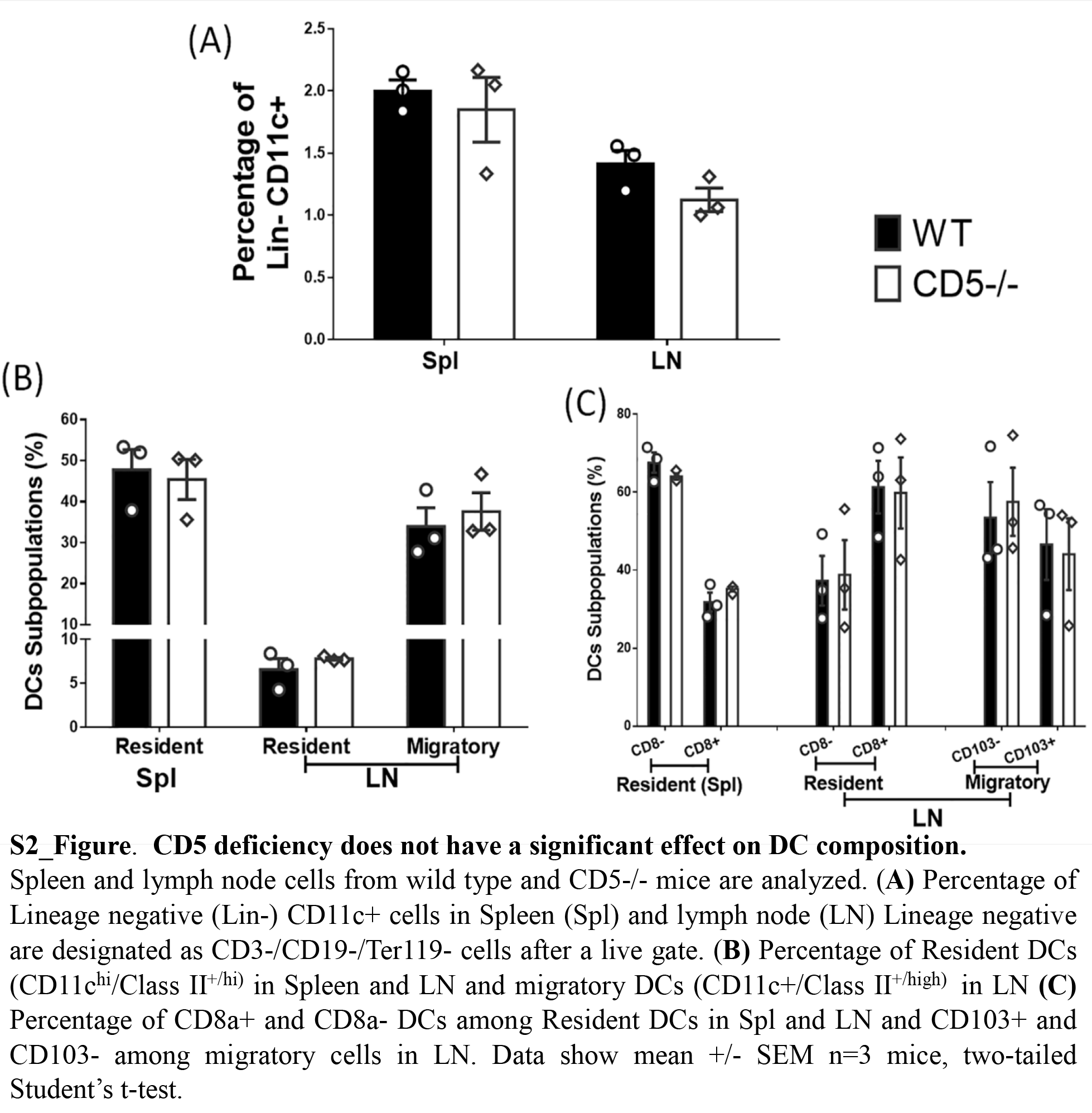

Supplement: S2 Fig — (TIF) [file pone.0222301.s002.tif]

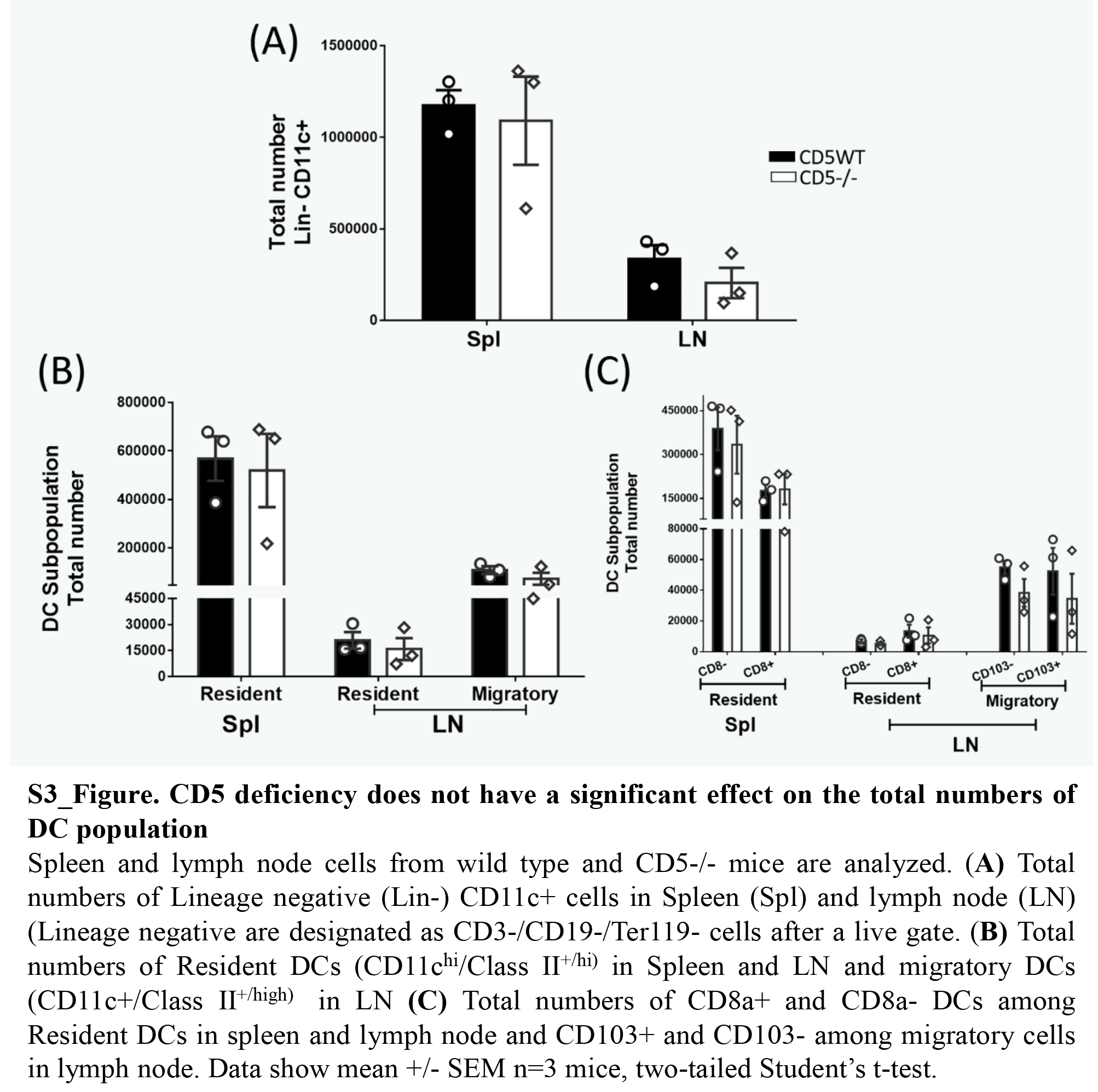

Supplement: S3 Fig — (TIF) [file pone.0222301.s003.tif]
